# P-2063. Who Gets What? Examining Demographic Influences on Antibiotic Prescriptions for Hospitalized Patients with Community-Acquired Pneumonia

**DOI:** 10.1093/ofid/ofaf695.2227

**Published:** 2026-01-11

**Authors:** Elizabeth Savage, Kellie Wark, Joanna Kimball, Kathryn Lamberton, Gabe Haas

**Affiliations:** University of Kansas School of Medicine, Overland Park, Kansas; University of Kansas, Kansas City, Kansas; The University of Kansas Medical Center, Kansas City, Kansas; The University of Kansas Health System, Shawnee, Kansas; Kansas Department of Health & Environment, Topeka, Kansas

## Abstract

**Background:**

The National Academy of Medicine recognizes equity as one of six healthcare quality domains, yet few studies have examined how antibiotic prescribing varies based on social determinants of health or provider characteristics. Data on inpatient prescribing are particularly limited, with no prior studies assessing differences in hospitalized patients with community acquired pneumonia (CAP). We investigated potential differences at a single academic center over four years.

**Figure:**
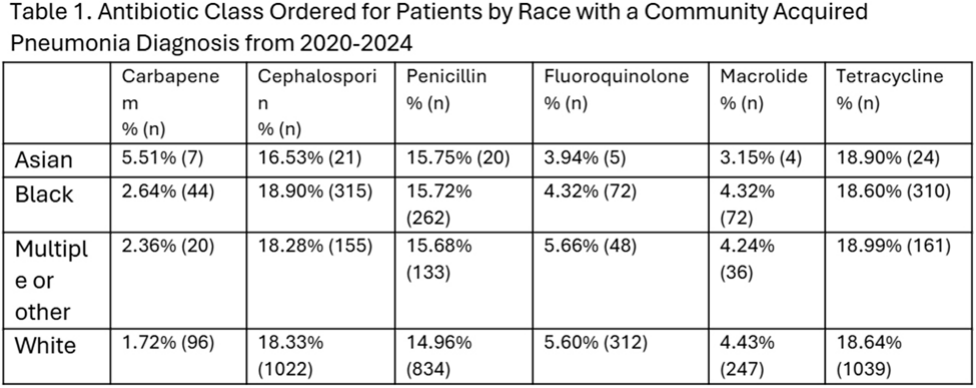

**Figure:**
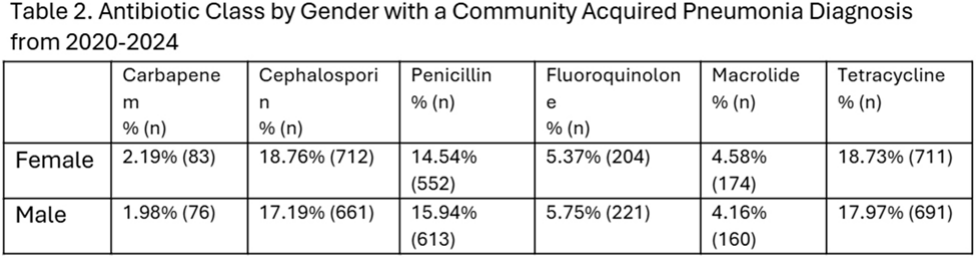

**Methods:**

This single-center, retrospective study included patients hospitalized with CAP at the University of Kansas Health System (2021-2024). The primary outcome was guideline-concordant antibiotic selection. Secondary variables included age, sex, gender, ethnicity, race, language, insurance status, route of antibiotic, social vulnerability index, and provider. Data were extracted from the electronic medical record (Epic) using Slicer Data and were analyzed in JASP.

**Figure:**
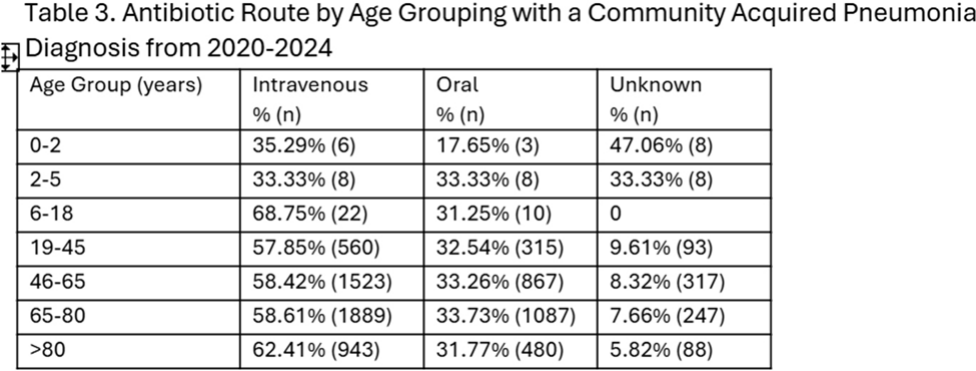

**Results:**

Cephalosporin use was similar among White (18.33%, n=1022), Black (18.90%, n=315), and Asian (16.53%, n=21) patients. Carbapenem use was highest among Asians (5.5%, n=7, p=0.0015) compared to Black (2.64%, n=44) and White (1.72%, n=96) patients. Penicillin prescribing was comparable between White (14.96%, n=834) and Black (15.72%, n=262) patients with lower rates in Asians (10.87%, n=5) (Table 1). Tetracycline use varied minimally across racial groups. When antibiotics were categorized into broad- versus narrow-spectrum, Black patients received broad-spectrum antibiotics slightly more frequently (38.33%, n=639) as compared to White patients (36.65%, n=2043; p=0.21). No significant differences were observed by gender (Table 2).

Route of administration was similar across racial groups: 58.78% in White, 58.55% in Black, and 59.10% in Asian patients. IV antibiotic use increased with age, with highest rates in patients > 80 (62.4%) and ages 6-18 (68.75%) (Table 3).

**Conclusion:**

While no significant disparities were identified, subtle differences merit further study. This study provides a framework for antimicrobial stewardship programs to monitor equity. Expanding this methodology across institutions and to other diagnoses could identify opportunities to improve equitable care.

**Disclosures:**

All Authors: No reported disclosures

